# Subclinical primary aldosteronism: toward a conceptual framework linking primary aldosteronism and low-renin hypertension

**DOI:** 10.3389/fendo.2026.1899618

**Published:** 2026-07-31

**Authors:** Chan Ho Park

**Affiliations:** Department of Internal Medicine, Dong-A University Hospital, Busan, Republic of Korea

**Keywords:** aldosterone-to-renin ratio, cardiovascular risk, mineralocorticoid receptor, primary aldosteronism, renin-independent aldosterone, subclinical aldosteronism

## Abstract

Primary aldosteronism (PA) has traditionally been considered a rare cause of secondary hypertension, but growing evidence supports a broader continuum of renin-independent aldosterone production extending into normotensive and mildly hypertensive populations. Subclinical primary aldosteronism (SPA) has emerged as a conceptual framework describing this milder phenotype. However, a universally accepted definition of SPA remains lacking, and consensus diagnostic criteria have yet to be established. This review synthesizes current evidence regarding SPA, including its epidemiology, pathophysiology, clinical implications, diagnostic challenges, and potential therapeutic strategies. Low-renin phenotypes are observed in a substantial proportion of individuals, although the true prevalence of SPA remains uncertain due to the lack of consensus diagnostic criteria. Observational studies have associated SPA with adverse cardiovascular and renal outcomes, including arterial stiffness, adverse cardiac remodeling, major adverse cardiovascular events, albuminuria, and chronic kidney disease progression, with effects not fully explained by blood pressure elevation alone. Conventional screening strategies based on the aldosterone-to-renin ratio have limitations in detecting this subclinical spectrum. Mineralocorticoid receptor antagonists and aldosterone synthase inhibitors represent hypothesis-generating therapeutic strategies, although prospective interventional evidence in SPA populations is lacking. At present, SPA should be regarded as an emerging risk-associated biochemical phenotype rather than an established disease category, pending prospective validation. Future studies are needed to determine whether consensus diagnostic criteria can be established and to validate clinically applicable screening strategies for this emerging endocrine phenotype.

## Introduction

Hypertension affects over 1.3 billion people worldwide and remains the leading modifiable risk factor for cardiovascular morbidity and mortality (1). Primary aldosteronism (PA)—characterized by autonomous, renin-independent aldosterone excess—is now recognized as one of the most common secondary causes of hypertension, affecting an estimated 5–10% of hypertensive patients in primary care and up to 21–30% in resistant hypertension (1–4).

**Figure 1:**
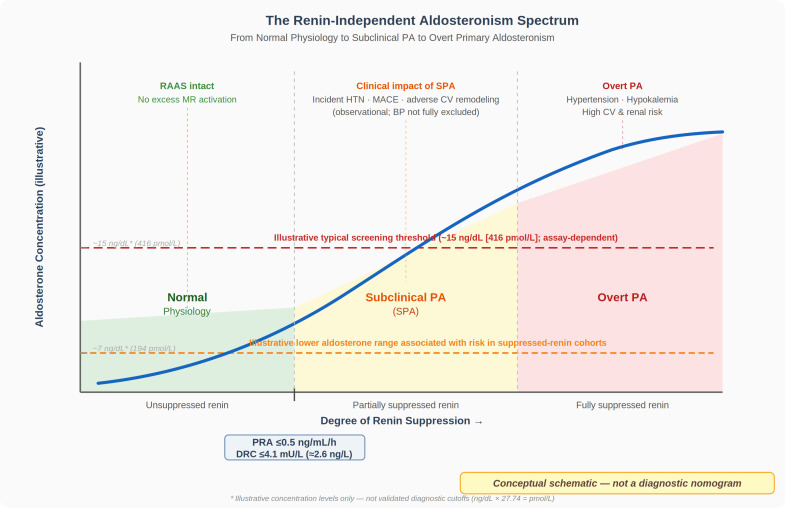
The renin-independent aldosteronism spectrum: from normal physiology to overt primary aldosteronism. Three color-coded zones, separated by vertical dashed lines, represent normal physiology (green; RAAS intact), subclinical PA (SPA, yellow; suppressed renin with inappropriately normal or mildly elevated aldosterone below PA diagnostic cutoffs, associated in observational studies with incident hypertension, adverse cardiovascular remodeling, and increased cardiovascular risk), and overt PA (red; meeting established diagnostic thresholds). The vertical zone boundary is marked at an illustrative renin suppression cutoff (PRA ≤0.5 ng/mL/h or DRC ≤4.1 mU/L (≈2.6 ng/L)), consistent with commonly used definitions in SPA epidemiology studies. All aldosterone concentrations are shown in ng/dL; SI equivalents: 7 ng/dL ≈ 194 pmol/L; 15 ng/dL ≈ 416 pmol/L. The dashed red line denotes the illustrative typical screening threshold range used in conjunction with elevated ARR for overt PA case detection (commonly used ARR range ≥31–70, assay-dependent; illustrative only). The dashed orange line indicates an illustrative lower aldosterone range associated with cardiovascular risk in suppressed-renin cohorts (approximately corresponding to ARR ≥12.5–25; ref. 8); this is not a validated diagnostic threshold. Y-axis values (~7 ng/dL and ~15 ng/dL) are illustrative approximations only and do not represent validated diagnostic cutoffs. The blue curve is a conceptual schematic of the aldosterone–renin relationship, not a diagnostic nomogram, and does not represent quantitative aldosterone levels. ARR, aldosterone-to-renin ratio; CKD, chronic kidney disease; HTN, hypertension; LVH, left ventricular hypertrophy; MACE, major adverse cardiovascular events; MR, mineralocorticoid receptor; RAAS, renin-angiotensin-aldosterone system; SPA, subclinical primary aldosteronism; PA, primary aldosteronism; BP, blood pressure.

More importantly, a growing body of evidence has illuminated a continuum of renin-independent aldosterone production that extends well beyond classical overt PA (5, 6). Population-based studies have demonstrated that low-renin phenotypes—characterized by suppressed plasma renin activity (PRA) in the absence of frank aldosterone excess—are prevalent even among normotensive individuals, and that aldosterone levels at or near the upper limits of the normal range carry excess cardiovascular and renal risk when renin is suppressed (7, 8). This milder end of the spectrum has been termed subclinical primary aldosteronism (SPA), a concept that challenges the binary diagnostic framework of PA and raises the prospect that a substantial proportion of the population may harbor clinically relevant mineralocorticoid receptor (MR) activation without meeting conventional diagnostic thresholds (5, 9). The proposed continuum from normal physiology through SPA to overt PA is illustrated schematically in **Figure 1**.

The clinical implications of SPA are far-reaching. Epidemiological data linking low-renin states to increased rates of incident hypertension, major adverse cardiovascular events (MACE), left ventricular hypertrophy (LVH), atrial fibrillation (AF), and chronic kidney disease (CKD) challenge clinicians and researchers to rethink screening paradigms and treatment strategies (8, 10, 11). Landmark studies in the CARTaGENE cohort published in Circulation and a subsequent longitudinal analysis have provided the most compelling population-level evidence to date that SPA may represent a clinically relevant cardiovascular risk-associated phenotype (7, 8).

Aldosterone-producing micronodules (APMs) are microscopic clusters of CYP11B2-positive aldosterone-producing cells within the adrenal cortex that autonomously produce aldosterone. Increasing evidence suggests that APMs accumulate with aging and may represent precursor lesions of aldosterone-producing adenomas, providing biological support for the continuum from SPA to overt PA (12).

This review synthesizes current evidence on SPA, covering its epidemiology, underlying pathophysiology—including the emerging role of aldosterone-producing micronodules (APMs)—clinical consequences, diagnostic challenges posed by ARR limitations, and evolving therapeutic strategies. The purpose of this review is not to establish formal diagnostic criteria for SPA, but rather to synthesize current evidence and propose a conceptual framework that may facilitate future consensus development. Recognition of SPA may have potential implications for hypertension screening strategies and early risk stratification in clinical practice. This review is a narrative synthesis of current evidence aimed at integrating epidemiological, mechanistic, and clinical data on subclinical primary aldosteronism, rather than a formal systematic review or meta-analysis.

## Methods

### Search strategy and study selection

This narrative review was conducted to synthesize current evidence on SPA and the broader spectrum of renin-independent aldosteronism. A focused literature search was performed using PubMed/MEDLINE up to March 2026. The following search terms were used in various combinations: “primary aldosteronism”, “subclinical aldosteronism”, “low-renin hypertension”, “aldosterone-to-renin ratio”, “renin-independent aldosterone”, “mineralocorticoid receptor”, and “cardiovascular outcomes”.

We included original research articles, cohort studies, clinical trials, and relevant review articles addressing epidemiology, pathophysiology, clinical outcomes, diagnosis, and treatment of PA and related subclinical phenotypes. Priority was given to large cohort studies, longitudinal analyses, and studies published in peer-reviewed journals. Studies were selected based on their clinical relevance, methodological quality, and contribution to understanding the continuum of renin-independent aldosterone excess. Additional references were identified through manual review of bibliographies of selected articles. Exclusion criteria included case reports, small case series with limited generalizability, non-English publications, and studies lacking sufficient methodological detail.

### Data extraction and synthesis

Data were extracted and qualitatively synthesized focusing on key domains: epidemiology, underlying mechanisms, clinical implications, diagnostic challenges, and therapeutic perspectives. Given the heterogeneity of study designs and the absence of standardized diagnostic criteria for SPA, a quantitative meta-analysis was not performed. This review follows a narrative synthesis approach and does not aim to provide pooled effect estimates but rather to integrate current evidence and highlight emerging concepts in the field. The search strategy was limited to human studies and articles published in English. Key publications were selected for qualitative synthesis based on relevance to the review objectives; additional references were identified through manual bibliography review. The search was not registered as a systematic review protocol (e.g., PROSPERO). No formal risk-of-bias assessment was performed, as this was a narrative review.

## Epidemiology of subclinical primary aldosteronism

### Prevalence of the low-renin phenotype

The epidemiological profile of SPA remains incompletely characterized, largely due to the absence of consensus diagnostic criteria and the heterogeneity of assay methods across studies. Nevertheless, accumulating data from several large cohort studies paint a consistent picture of a highly prevalent but long-overlooked condition. Recent systematic evidence underscores the high burden of the low-renin phenotype: Chauhan et al. conducted a meta-analysis of 60 studies encompassing 11,781 participants and reported a pooled prevalence of low-renin hypertension of 35% (95% CI 32–38%), establishing it as one of the most common biochemical phenotypes among hypertensive individuals (13).

Shah et al. examined 261 treatment-naïve hypertensive patients in a primary care setting and found that 26.4% met criteria for low-renin hypertension (direct renin concentration <10 mU/L, equivalent to approximately 6.34 ng/L and corresponding to a PRA of approximately 1.22 ng/mL/h), while 18.0% had confirmed PA (14). Notably, Brown et al. demonstrated a graded increase in the prevalence of biochemically overt PA across the blood pressure continuum: from 11.3% among normotensive individuals to 15.7% in stage 1 hypertension, 21.6% in stage 2 hypertension, and 22.0% in resistant hypertension—further supporting the concept that renin-independent aldosterone excess represents a continuum rather than a binary diagnostic entity (15). Notably, those with low-renin hypertension had median aldosterone concentrations lower than patients with frank PA but higher than those with normal-renin hypertension, consistent with an intermediate, subclinical phenotype. In the landmark Multi-Ethnic Study of Atherosclerosis (MESA), Brown et al. demonstrated that 850 normotensive participants with suppressed PRA (≤0.50 ng/mL/h) had significantly higher rates of incident hypertension over follow-up, and that higher aldosterone concentrations within this suppressed-renin cohort conferred additive risk—suggesting a spectrum of renin-independent aldosteronism even before hypertension develops (9).

The CARTaGENE cohort study, a population-based sample of 1,284 adults aged 41–69 years in Québec, Canada, provided foundational evidence for SPA’s cardiovascular relevance (7, 16). Renin and aldosterone were measured and participants were stratified by aldosterone-to-renin ratio (ARR) and renin level. A threshold analysis further identified that DRC ≤4.0 ng/L (equivalent to approximately 6.31 mU/L and corresponding to a PRA of approximately 0.77 ng/mL/h) and an ARR ≥70 pmol/L per ng/L (equivalent to approximately 13.14 ng/dL per ng/mL/h) were associated with a 2.4-fold higher risk of MACE in a subsequent longitudinal analysis—importantly, below the traditional diagnostic threshold for PA (8). These findings support the plausibility of a clinically relevant subclinical spectrum. Key epidemiological studies characterizing SPA and related low-renin phenotypes are summarized in **Table 1**. A comparative summary of the renin, aldosterone, and ARR criteria used across these studies is presented in **Supplementary Table 1**.

**Table 1:** Key epidemiological studies of subclinical and low-renin primary aldosteronism.

Study (Year)	Population	Design	Key Finding
Brown et al. (9) Ann Intern Med	850 normotensive adults (MESA cohort)	Cohort study	Suppressed PRA associated with 85.4/1,000 PY incidence of HTN vs. 54.5 (intermediate-renin) and 53.3 (high-renin) per 1,000 PY; aldosterone independently associated with HTN within suppressed-renin group (ref 9)
Hundemer et al. (7) Circulation	1,284 adults aged 41–69 years (CARTaGENE cohort)	Prospective cohort	Subclinical PA associated with arterial stiffness (PWV β = +0.33 m/s), elevated LVMI (β = +5.1 g/m^2^), and LVH after multivariable adjustment including BP (ref 7)
Goupil et al. (8) Circulation	CARTaGENE cohort, adults aged 41–69 years	Longitudinal cohort	ARR ≥70 pmol/L per ng/L + renin ≤4 ng/L → HR 2.4 for MACE (95% CI 1.4–4.2); effect persisted after BP adjustment; below PA diagnostic threshold (ref 8)
Shah et al. (14) J Endocr Soc	261 treatment-naïve hypertensives (primary care)	Cohort study	26.4% low-renin hypertension; 18.0% confirmed PA; intermediate aldosterone phenotype in SPA group (ref 13)
Markou et al. (16) J Clin Endocrinol Metab	Predominantly normotensive cohort	Cross-sectional/longitudinal	OR 12.7 for progression to HTN at 7-year follow-up in normotensives meeting PA biochemical criteria (ref 15)

### Prevalence across the blood pressure continuum

A critical distinguishing feature of SPA is its presence across the entire blood pressure spectrum. Markou et al. described a cohort of predominantly normotensive individuals fulfilling PA biochemical criteria, who had a high odds ratio for progression to arterial hypertension (17). The concept of nonclassic PA was further elaborated by Buffolo et al. in their description of the spectrum of low-renin hypertension (18).

Contemporary reviews have reinforced that PA and its subclinical forms are detectable across the full continuum of blood pressure severity—from normotension to resistant hypertension—and that reliance solely on hypertension-based screening criteria leads to systematic under-detection (19). Collectively, these data suggest that the true prevalence of SPA in the general adult population is likely underestimated, though firm prevalence figures await the establishment of consensus diagnostic criteria.

## Pathophysiology

### Renin suppression as the fundamental feature

Renin suppression is the defining biochemical hallmark of the renin-independent aldosteronism spectrum. In PA and SPA, the normal RAAS negative feedback is bypassed: aldosterone secretion continues autonomously, maintaining renin suppression while driving sodium retention and MR activation (2, 5). In SPA, this autonomy is partial; aldosterone levels remain inappropriately elevated relative to suppressed renin—a concept termed “relative aldosterone excess”—without necessarily crossing traditional PA diagnostic thresholds (5, 9). Physiologically, SPA may be distinguished from low-renin essential hypertension by partial aldosterone autonomy, reflected by impaired suppression in dynamic testing, although this distinction remains incompletely defined. Parisien-La Salle et al. further characterized this boundary using natriuretic peptide responses, ACTH stimulation, and angiotensin II sensitivity testing, demonstrating differential adrenal responsiveness that may help biologically separate SPA from low-renin essential hypertension in research settings (20). These findings reinforce that renin suppression alone is insufficient to diagnose SPA, and functional testing may be required for phenotypic disambiguation.

### Aldosterone-producing micronodules as plausible cellular substrate

APMs—small clusters of CYP11B2-immunopositive cells beneath the adrenal capsule—have been proposed as a plausible cellular substrate for subclinical aldosterone excess (21–24). APMs frequently harbor somatic driver mutations (notably CACNA1D) distinct from those in overt PA adenomas, and their cumulative adrenal burden may determine the degree of autonomous aldosterone excess (23, 25, 26).

### Molecular mechanisms of autonomous aldosterone production

Known driver mutations—including KCNJ5, CACNA1D, ATP1A1, and ATP2B3—converge on a common pathway of increased intracellular Ca^2+^ signaling that activates CYP11B2 transcription (26–29). Additional contributors include zona glomerulosa hyperplasia and increased ACTH sensitivity (24). The cumulative burden of these molecular abnormalities across the adrenal cortex likely determines the degree of aldosterone excess and position along the subclinical-to-overt spectrum. Recent comprehensive reviews by Aminuddin et al. and Itcho et al. have further delineated how adrenal cortical cell differentiation, CYP11B2 transcriptional regulation, and these somatic driver mutations—including the less common CACNA1H, CLCN2, and CTNNB1 variants—collectively determine the degree of autonomous aldosterone production, providing molecular grounding for the clinical continuum from SPA to overt PA (30, 31).

### MR activation and tissue effects

Aldosterone exerts its pathological effects primarily through the MR, a nuclear receptor that modulates gene transcription. In epithelial tissues, particularly the distal nephron and collecting duct, MR activation drives sodium reabsorption and potassium secretion, contributing to volume expansion and hypertension (2, 5). However, MR is also widely expressed in non-epithelial tissues including the heart, vasculature, brain, and immune cells, where its activation promotes inflammation, oxidative stress, fibrosis, and vascular remodeling through pathways involving transforming growth factor-β (TGF-β), reactive oxygen species (ROS), and connective tissue growth factor (CTGF) (3, 5).

Critically, these non-hemodynamic effects of MR activation appear to occur at aldosterone levels that do not cause overt hypertension or hypokalemia (5, 7). In contrast to overt PA, where hypokalemia is present in approximately 41–50% of patients, patients with SPA characteristically lack significant hypokalemia, reflecting the milder degree of autonomous aldosterone excess. This is consistent with the growing body of observational evidence demonstrating target organ damage—including increased LV mass, arterial stiffness, and albuminuria—in patients with SPA whose blood pressure is only mildly elevated or within the normal range, although residual confounding by unmeasured blood pressure–related factors cannot be excluded in existing cohort studies (7, 8, 10, 32).

## Clinical implications of subclinical primary aldosteronism

### Is SPA predominantly bilateral?

A clinically important but underexplored question concerns the laterality of SPA. Available evidence suggests that SPA predominantly represents a bilateral phenotype: the cellular substrate most implicated—diffuse zona glomerulosa hyperplasia and multiple APMs accumulating with age—implies a distributed, gland-wide process rather than a focal lesion (21–24). This contrasts with overt PA, in which lateralized adenomas account for a substantial proportion of confirmed cases. Whether SPA predominantly reflects an early bilateral form of aldosterone dysregulation, or whether a subset of individuals represents an early stage of future lateralized PA, remains unknown. Longitudinal evidence supporting progression from SPA to overt PA is limited; however, studies by Brown et al. and Markou et al. suggest that autonomous aldosterone secretion may evolve over time, supporting a biological continuum hypothesis (9, 17). Importantly, Buffolo et al. demonstrated that patients with elevated ARR but negative confirmatory testing may progress toward overt PA phenotypes during long-term follow-up, providing additional longitudinal support for the biological continuum hypothesis (33). If SPA is predominantly bilateral, adrenalectomy would not be an appropriate target, and MR antagonism would represent the principal therapeutic avenue. Future longitudinal studies with serial adrenal imaging and functional testing will be needed to clarify the natural history and laterality of SPA.

### Cardiovascular consequences

#### Structural cardiac remodeling

**Figure 2:**
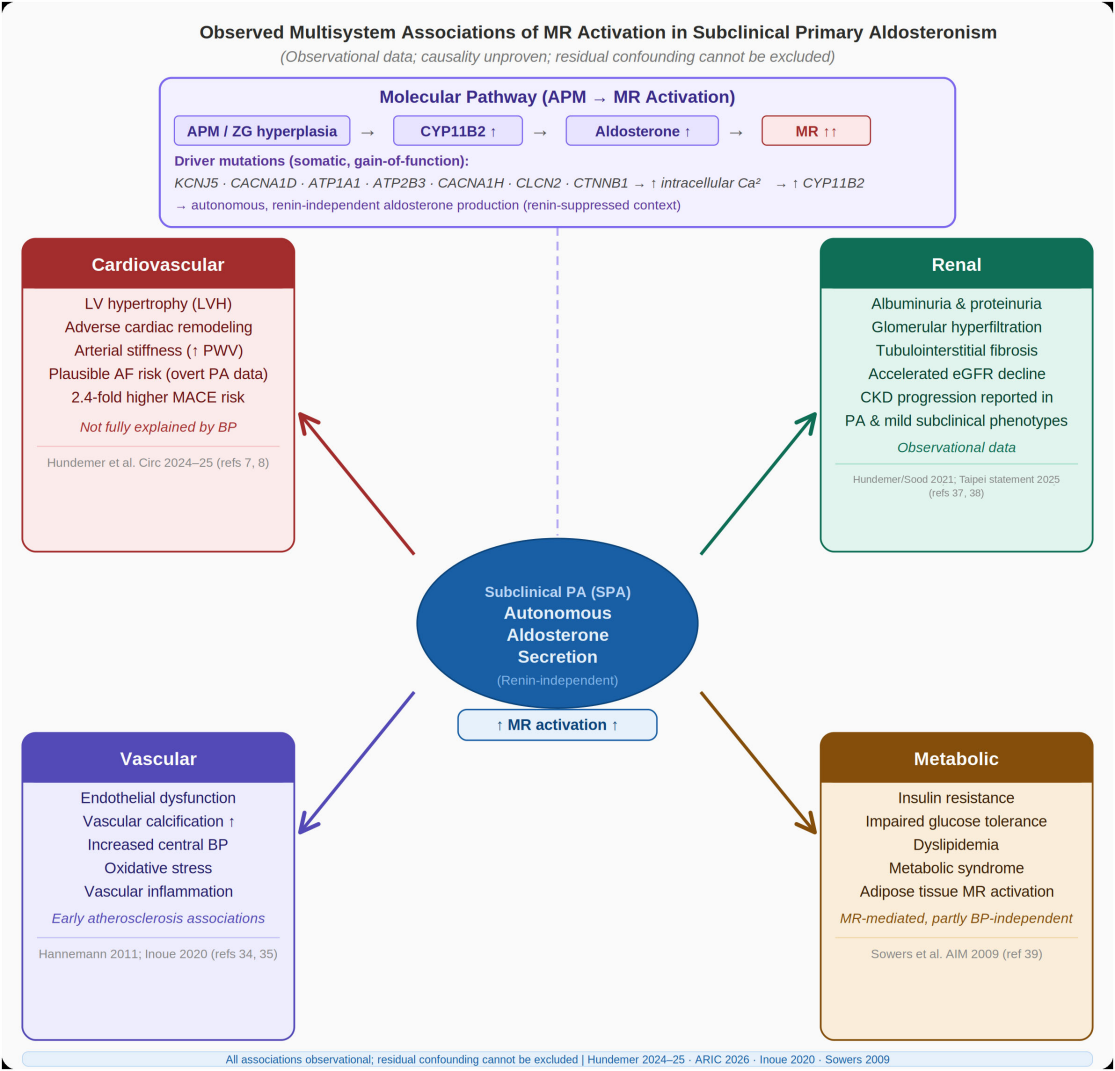
Observed cardiovascular, renal, vascular, and metabolic associations of subclinical primary aldosteronism. Upper inset: simplified molecular pathway from aldosterone-producing micronodules (APMs) through CYP11B2 activation (driven by somatic gain-of-function mutations in KCNJ5, CACNA1D, ATP1A1, ATP2B3, CACNA1H, CLCN2, and CTNNB1) to autonomous aldosterone production and MR activation. The central hub (Autonomous Aldosterone Secretion, Renin-Independent) drives MR activation across four organ systems (color-coded arrows). Cardiovascular (red): LVH, adverse cardiac remodeling, arterial stiffness, plausible AF risk continuum based on overt PA data, and 2.4-fold higher observed MACE risk after multivariable adjustment for BP (7, 8). Renal (green): albuminuria, glomerular hyperfiltration, tubulointerstitial fibrosis, accelerated eGFR decline, and CKD progression reported in PA and mild/subclinical phenotypes (37, 38). Vascular (purple): endothelial dysfunction, vascular calcification, oxidative stress, early atherosclerosis (34, 35). Metabolic (orange): insulin resistance, impaired glucose tolerance, dyslipidemia, and metabolic syndrome via MR-mediated pathways (39). Effects across all domains are not fully explained by BP elevation alone; residual confounding cannot be excluded. MR, mineralocorticoid receptor; LVH, left ventricular hypertrophy; AF, atrial fibrillation; MACE, major adverse cardiovascular events; eGFR, estimated glomerular filtration rate; CKD, chronic kidney disease; BP, blood pressure.

The observed multisystem associations potentially related to aldosterone-mediated MR activation in SPA, spanning cardiovascular, renal, vascular, and metabolic domains, are summarized in **Figure 2**. It is important to note that much of the quantitative outcome data in this section derives from the CARTaGENE cohort; independent replication in distinct populations is required before these associations can be considered generalizable. Landmark evidence from the CARTaGENE prospective cohort demonstrated that subclinical renin-independent aldosteronism is independently associated with multiple markers of adverse cardiac remodeling as assessed by cardiac magnetic resonance imaging (CMR). Participants with low renin and higher ARR exhibited significantly increased left ventricular mass index (LVMI), left ventricular remodeling index, indexed maximum left atrial volume, and higher rates of LVH—associations that remained significant after multivariable adjustment for blood pressure level, dietary sodium intake, age, and other confounders (7). These findings are consistent with direct aldosterone-mediated cardiac injury that may begin before hypertension becomes clinically apparent, though residual confounding cannot be entirely excluded in observational data.

#### Arterial stiffness and endothelial dysfunction

Arterial stiffness, assessed by pulse wave velocity (PWV) and central blood pressure, was significantly elevated in SPA participants in the CARTaGENE cohort (7). Earlier cross-sectional data from Hannemann et al. demonstrated associations between plasma aldosterone and ARR with endothelial dysfunction assessed by flow-mediated dilation in young-to-middle-aged subjects (34). More recent studies confirm that even modestly elevated aldosterone in the setting of renin suppression is associated with increased vascular calcification—a marker of advanced atherosclerosis (35).

#### Major adverse cardiovascular events

The most compelling evidence for SPA’s clinical impact comes from a recent longitudinal analysis of the CARTaGENE cohort. This study demonstrated that renin ≤4.0 ng/L combined with ARR ≥70 pmol/L per ng/L—a threshold substantially below the conventional PA diagnostic cutoff—was associated with a 2.4-fold higher risk of incident MACE, defined as the composite of myocardial infarction, stroke, hospitalization for heart failure, and cardiovascular death, in observational analyses. These associations remained significant after multivariable adjustment for blood pressure, dietary sodium, age, sex, and metabolic risk factors, though as with all observational data, residual confounding cannot be fully excluded (8). The accompanying editorial characterized SPA as a “potential new target in cardiovascular prevention” (6).

#### Atrial fibrillation and heart failure

Overt PA is well-established as a risk factor for AF and HF, with a meta-analysis by Monticone et al. demonstrating significantly higher rates of these events compared to blood-pressure-matched essential hypertension (10). Although direct evidence in SPA is more limited, mechanistic data on atrial fibrosis driven by MR activation and overt PA cohort data (10, 11) support a plausible continuum of AF risk extending into the subclinical range; prospective SPA-specific data on AF incidence are currently lacking.

Recent data from the Atherosclerosis Risk in Communities (ARIC) cohort provide important nuance to the cardiovascular risk profile of low-renin aldosteronism (36). In the ARIC study, low-renin states were associated with higher risk of AF and stroke but not with heart failure or myocardial infarction, suggesting that aldosterone-mediated cardiovascular effects may be organ-selective rather than uniformly adverse across all cardiovascular endpoints. These findings support the biological plausibility of an SPA-cardiovascular risk association while underscoring the need for outcome-specific analyses and well-defined endpoint selection in future SPA studies, rather than reliance on composite MACE endpoints alone.

### Renal consequences

#### Albuminuria and glomerular injury

Aldosterone-mediated glomerular hyperfiltration, mesangial expansion, and podocyte injury contribute to proteinuria and progressive nephron loss (21, 37). Even in the setting of subclinical aldosterone excess, increased albuminuria has been observed in multiple cohorts. The 2025 Taipei Positional Paper on universal PA screening summarized consistent observational data demonstrating increased CKD prevalence, proteinuria, and more rapid estimated glomerular filtration rate (eGFR) decline in patients with PA compared to renin-appropriate hypertensive controls, with the authors noting that these risks persist in individuals with subclinical or mild PA not meeting traditional diagnostic thresholds (38).

#### CKD progression

Aldosterone drives renal fibrosis through MR-mediated TGF-β and CTGF upregulation, resulting in tubulointerstitial damage and glomerulosclerosis (37, 39). Subclinical MR activation may therefore contribute to CKD progression through mechanisms not fully explained by blood pressure elevation alone. Importantly, there is bidirectional interplay: CKD-related volume expansion may further suppress renin and amplify the relative inappropriateness of aldosterone levels, potentially creating a vicious cycle of cardiorenal deterioration (37).

### Incident hypertension

Perhaps the most immediately actionable implication of SPA is its role as a precursor to hypertension. A MESA cohort study demonstrated that normotensive adults with suppressed PRA developed incident hypertension at a rate of 85.4 per 1,000 person-years—compared with 54.5 and 53.3 per 1,000 person-years in the intermediate- and high-renin groups respectively—highlighting a stepwise gradient of risk across the renin spectrum (9). Within the suppressed-renin group, higher aldosterone concentrations—including levels in the upper-normal range—were independently associated with higher hypertension incidence, providing direct evidence for aldosterone-driven hypertension development at levels below the traditional PA diagnostic threshold (9, 18).

### Metabolic consequences

Aldosterone has been implicated in insulin resistance and metabolic dysregulation through MR-mediated mechanisms in adipose tissue, skeletal muscle, and the pancreas, through pathways that are largely independent of its hemodynamic effects (39). Subclinical MR activation may therefore contribute to the cardiometabolic risk cluster observed in low-renin populations, including impaired glucose tolerance, dyslipidemia, and metabolic syndrome—further broadening the clinical relevance of SPA beyond hypertension and overt cardiovascular disease.

## Defining SPA: current challenges and proposed framework

### Limitations of the aldosterone-to-renin ratio

The ARR remains the cornerstone of PA screening and is recommended as the first-line case-detection test by all major guidelines, including the 2024 ESC Hypertension Guidelines (Class IIa, Level B) and recent Endocrine Society guideline updates (40–42). For overt PA, the ARR has demonstrated sensitivity up to 97.7% and a negative predictive value of 99% in optimized conditions (43). However, the ARR has fundamental limitations that render it poorly suited to capturing the subclinical spectrum.

First, the ARR is mathematically dominated by renin when renin values are extremely low: minor absolute differences in near-zero renin concentrations produce disproportionately large ARR fluctuations, making it highly susceptible to assay variability. Second, no standardized ARR cutoff exists across assay platforms: published cutoffs range from 20 to 100 depending on whether direct renin concentration (DRC) or PRA is used, the laboratory method, and the units applied (43). Third, numerous medications influence the ARR—mineralocorticoid receptor antagonists, ACE inhibitors, ARBs, beta-blockers, diuretics, and oral contraceptives all affect renin or aldosterone to varying degrees (40).

Perhaps most critically for SPA, the principal diagnostic limitation may be the requirement for a minimum absolute aldosterone threshold rather than failure of ARR elevation itself. In many individuals with SPA, renin is already markedly suppressed; the resulting ARR may in fact be elevated despite only modest absolute aldosterone concentrations. The diagnostic bottleneck is therefore the aldosterone floor—patients with aldosterone levels below conventional minima (e.g., <10–15 ng/dL (277–416 pmol/L)) are excluded from PA confirmation regardless of ARR, leading to systematic under-detection of the SPA phenotype (5, 9).

### The 2025 endocrine society guidelines: toward simplified diagnosis

The revised 2025 Endocrine Society Clinical Practice Guideline for Primary Aldosteronism represents a major paradigm shift in PA diagnosis (42). Critically, it moves toward simplified confirmation pathways and endorses universal screening of all hypertensive patients—not only those with hypokalemia or resistant hypertension—thereby dramatically broadening the population eligible for case detection. A key update is the recognition that confirmatory testing may be waived in patients with a characteristic biochemical triad of suppressed renin, elevated aldosterone, and elevated ARR when pretest probability is high and the biochemical phenotype is strongly positive, streamlining diagnosis for appropriately identified cases. The guideline also acknowledges the concept of renin-independent aldosterone excess as a spectrum, and emerging analyses suggest the 2025 criteria may substantially increase PA detection rates versus 2016. Crucially, by embracing renin-first phenotyping and a spectrum model, these guidelines create an important conceptual bridge to the SPA construct; extending criteria to capture subclinical phenotypes will require further evolution—likely incorporating renin phenotyping as a primary criterion rather than a secondary confirmation tool. Similarly, recent hypertension guidelines have expanded PA screening recommendations, particularly in patients with resistant hypertension and stage 2 hypertension, further broadening the population in which renin-independent aldosterone excess may be identified. The 2025 guideline provides assay- and unit-specific screening thresholds for PRA, DRC, aldosterone concentration, ARR, and ADRR, thereby facilitating more standardized interpretation and comparison across clinical settings. Throughout this review, guideline-recommended thresholds are presented where appropriate, whereas the original assay-specific definitions are retained when individual observational studies are described.

### Age-specific and assay-specific considerations

ARR thresholds exhibit significant age-dependence: optimal cutoffs for patients under 40 years differ substantially from those for patients over 60 years, reflecting physiological changes in renin secretion with aging. A threshold of ADRR 3.7 ng/dL per mU/L (equivalent to approximately 30.34 ng/dL per ng/mL/h) was optimal for patients ≥60 years, whereas a cutoff of 1.0 ng/dL per mU/L (equivalent to approximately 8.2 ng/dL per ng/mL/h) may be more appropriate for patients under 40—a more than threefold difference (44). These age-related variations further complicate the application of uniform ARR cutoffs to SPA screening.

### Proposed diagnostic approach for SPA

Given these limitations, a complementary diagnostic approach incorporating renin phenotyping as a primary criterion—rather than relying solely on aldosterone and ARR—may represent a useful strategy for capturing the SPA spectrum in selected clinical contexts (5, 9). Specifically, PRA ≤0.5 ng/mL/h or DRC ≤4.1 mU/L (≈2.6 ng/L) may be considered a complementary screening approach, with the aldosterone concentration and ARR providing secondary stratification. Within the suppressed-renin phenotype, aldosterone concentrations in the upper-normal range (e.g., >7 ng/dL (194 pmol/L)) may warrant heightened clinical attention even without meeting traditional PA thresholds. It should be emphasized that this approach is exploratory and not currently supported by major clinical guidelines; prospective validation in diverse populations is required before any recommendation can be made. These values should be interpreted as illustrative ranges intended for research use only, rather than proposed diagnostic thresholds for clinical decision-making. Standardization of renin assays and aldosterone measurements across laboratories remains an essential prerequisite for implementing such an approach at scale (43). From a clinical perspective, reliance on ARR alone may lead to under-recognition of a substantial proportion of low-renin patients at increased cardiovascular risk. Incorporating renin phenotyping into routine hypertension evaluation may therefore represent a pragmatic and clinically relevant approach. At present, SPA should be interpreted as a risk-associated biochemical phenotype rather than a distinct disease entity with established diagnostic and therapeutic pathways. A comparison of key diagnostic features between overt PA and the proposed subclinical PA phenotype is presented in **Table 2**. A conceptual research framework for identifying individuals with possible SPA, intended to guide future research design rather than clinical decision-making, is presented in **Table 3**. Current areas of agreement and ongoing controversy in the SPA field are summarized in **Table 4**.

**Table 2:** Comparison of overt PA and proposed subclinical PA phenotypes.

Feature	Overt PA	Subclinical PA (Proposed)
Renin level	Suppressed (PRA <1 ng/mL/h or DRC <8.2 mU/L (5.2 ng/L))	Frequently reported suppressed-renin range, e.g., PRA ≤0.5 ng/mL/h or DRC ≤4.1 mU/L (2.6 ng/L)
Aldosterone	Elevated (>15 ng/dL (416 pmol/L))	Upper-normal aldosterone range reported in observational studies (e.g., >7 ng/dL (194 pmol/L))
ARR	>20 ng/dL per ng/mL/h or aldosterone-to-DRC ratio (ADRR) >70 pmol/L per mU/L	Observational risk-associated range, e.g., ARR >12.5 ng/dL per ng/mL/h or ADRR >42 pmol/L per mU/L
Hypokalemia	Common (41–50%)	Absent or mild
Hypertension	Typical feature	May be absent (normotensive to mildly hypertensive)
Confirmatory testing	Usually required; may be omitted in selected patients with overt biochemical phenotype (ES 2025)	Not established; may not be applicable
Guideline recognition	Recognized in major PA/hypertension guidelines (ES 2025, ESC 2024)	Emerging; not yet in standard guidelines

Proposed SPA thresholds are exploratory and are not intended as validated diagnostic criteria, and should not be used for clinical decision-making; prospective validation across diverse clinical settings is required before clinical application.

**Table 3:** Conceptual research framework for identifying individuals with possible SPA.

Domain	Suggested feature for SPA research identification
Blood pressure	Hypertension or elevated BP/prehypertension (according to applicable guideline definitions; not required — may be absent in normotensives)
Renin	Frequently reported suppressed range, e.g., PRA ≤0.5 ng/mL/h or DRC ≤4.1 mU/L (2.6 ng/L)
Aldosterone	High-normal aldosterone level, e.g., >7 ng/dL (>194 pmol/L)
ARR	Elevated below the traditional PA threshold; e.g., ARR >12.5 ng/dL per ng/mL/h or ADRR >42 pmol/L per mU/L
Overt PA criteria	Not met (below conventional PA diagnostic threshold)
Confirmatory testing	Negative or borderline; aldosterone not fully suppressed in dynamic testing

This framework is intended to guide future research design and hypothesis generation, not clinical decision-making. All thresholds are illustrative and require prospective validation. ARR, aldosterone-to-renin ratio; DRC, direct renin concentration; PA, primary aldosteronism; PRA, plasma renin activity; SPA, subclinical primary aldosteronism.

**Table 4:** Current areas of agreement and controversy in subclinical primary aldosteronism.

Areas of agreement	Areas of controversy
Renin suppression is a central biochemical feature of SPA	No consensus definition of SPA exists across studies
SPA is associated with adverse CV outcomes in observational cohorts	Causality unproven; residual confounding cannot be excluded
ARR and aldosterone thresholds require standardization	Optimal ARR/aldosterone cutoffs for SPA remain undefined
MR antagonists are biologically plausible in SPA	MRA treatment in SPA remains investigational; no RCT evidence
APMs provide a plausible cellular substrate	Bilateral vs. evolving lateralized nature of SPA unclear
A biological continuum from SPA to overt PA is plausible	Whether SPA is a disease entity or risk phenotype is debated

## Therapeutic implications

### Mineralocorticoid receptor antagonists

MRAs are the pharmacological cornerstone of treatment for overt bilateral PA and are used adjunctively in lateralized PA prior to adrenalectomy. Their potential role in SPA has been hypothesized to represent one of the most important unanswered questions in the field (5, 6, 45, 46).

Three generations of MRAs are available: spironolactone (first-generation; potent but with hormonal side effects), eplerenone (second-generation; more selective, less potent), and finerenone (third-generation, non-steroidal; favorable tolerability in large cardiorenal trials including FIDELIO-DKD and FIGARO-DKD; emerging evidence in early cardiovascular-kidney-metabolic stages) (47–51). For overt PA, spironolactone or eplerenone are established; finerenone’s role in PA is emerging (52).

### Finerenone in primary aldosteronism and subclinical disease

Hu et al. published a pilot randomized controlled trial demonstrating that finerenone reduced blood pressure in PA patients with acceptable tolerability, providing the first controlled evidence for its use in PA (52). Uslar et al. subsequently reported real-world finerenone outcomes in overt PA, noting clinically meaningful blood pressure reduction with a favorable safety profile (53). Importantly, Li et al. (54) conducted a systematic review concluding that while both steroidal and non-steroidal MRAs are effective in PA, head-to-head comparative data remain limited; finerenone cannot yet be recommended preferentially over established agents without adequately powered trials (54). The cardiorenal profile of finerenone—demonstrated in large RCTs in CKD and type 2 diabetes (FIDELIO-DKD, FIGARO-DKD)—provides mechanistic rationale for MR blockade in states of aldosterone excess (47–49). However, it is important to emphasize that these trial data were obtained in patients with diabetes and CKD, not in SPA populations. The potential applicability of finerenone to SPA is currently hypothesis-generating: the biological rationale is compelling, but direct evidence from interventional studies in subclinical or normotensive populations is entirely absent.

### Early intervention concept and the SPA treatment hypothesis

The concept of early MRA intervention in SPA rests on two premises: (1) that aldosterone-mediated target organ damage accrues cumulatively and may be partially reversible with early treatment, and (2) that the cardiovascular and renal events associated with SPA (MACE, CKD progression, LVH, AF) are preventable. Although this hypothesis is biologically plausible—supported by the PATHWAY-2 trial showing that spironolactone was the most effective add-on antihypertensive in resistant hypertension among patients with the lowest renin levels (19)—prospective interventional data in normotensive or mildly hypertensive SPA populations are entirely lacking.

**Figure 3:**
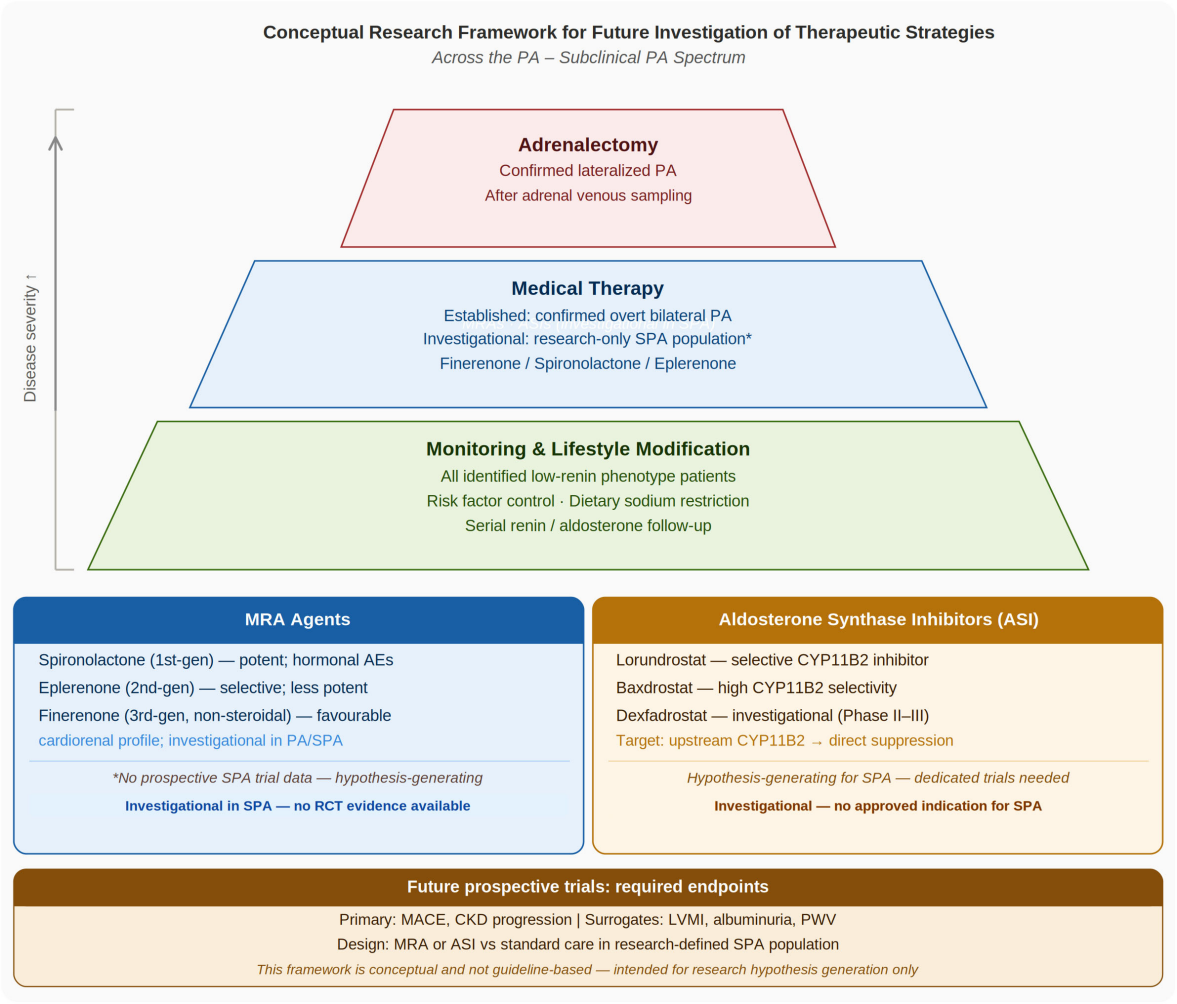
Conceptual research framework for future investigation of therapeutic strategies across the PA–subclinical PA spectrum. This framework is conceptual and not guideline-based. A three-tiered pyramid stratified by disease severity. Base tier (green): monitoring, lifestyle modification, and serial renin/aldosterone follow-up, applicable to all identified low-renin/SPA patients. Middle tier (blue): medical therapy, comprising mineralocorticoid receptor antagonists (MRAs) and emerging aldosterone synthase inhibitors (ASIs). MRAs are established therapy for confirmed overt bilateral PA, whereas both MRA and ASI treatment remain investigational in research-defined SPA populations, with no treatment recommendation implied (*). Apex tier (red): adrenalectomy for confirmed lateralized PA after adrenal venous sampling. Lower panels summarize available MRA agents and emerging therapeutic evidence (47, 48, 49–54) alongside emerging aldosterone synthase inhibitors (ASIs: including lorundrostat, baxdrostat, and dexfadrostat) (55), all investigational in SPA, and trial design priorities. (*) No prospective MRA or ASI trial data are available specifically in SPA; all proposed applications in SPA are hypothesis-generating. MRA, mineralocorticoid receptor antagonist; SPA, subclinical primary aldosteronism; PA, primary aldosteronism; MACE, major adverse cardiovascular events; CKD, chronic kidney disease; LVMI, left ventricular mass index; PWV, pulse wave velocity.

A key challenge is the risk-benefit calculus: MRAs carry real risks of hyperkalemia, particularly in patients with CKD or concurrent RAAS blockade, and spironolactone/eplerenone carry hormonal side effects. Treatment of large subclinical populations would require highly favorable safety profiles, clear identification of who will benefit, and validated surrogate endpoints. Finerenone may represent a potential candidate for future investigation in SPA, given its non-steroidal MR-selective profile and favorable tolerability observed in large cardiorenal outcome trials (47, 48, 53). A conceptual framework for therapeutic intervention across the PA–SPA spectrum, stratified by disease severity, is presented in **Figure 3**.

### Emerging aldosterone synthase inhibitors

A novel pharmacological strategy targeting the aldosterone biosynthetic pathway has emerged as a promising complement to MR antagonism. Aldosterone synthase inhibitors (ASIs) directly suppress CYP11B2-mediated aldosterone synthesis, potentially offering more upstream control of autonomous aldosterone production than MR blockade. Several aldosterone synthase inhibitors, including lorundrostat, baxdrostat, and dexfadrostat, are currently under clinical investigation. In the Target-HTN randomized trial, lorundrostat significantly reduced blood pressure and suppressed aldosterone in patients with uncontrolled hypertension, with acceptable effects on cortisol synthesis at therapeutic doses (55). Baxdrostat and dexfadrostat are at similar early stages of clinical investigation, with emerging data from phase II trials in resistant hypertension. In the context of SPA, ASIs represent a particularly compelling investigational strategy: by reducing aldosterone production at its source, they could theoretically interrupt the pathological cascade before overt target organ damage develops. However, their applicability to SPA remains entirely hypothesis-generating; no trials have evaluated ASIs specifically in subclinical or low-renin aldosteronism populations. Whether ASIs might prove superior, complementary, or equivalent to MRAs in SPA will require dedicated prospective trials with pre-specified SPA definitions and hard cardiorenal endpoints.

## Future directions

### Toward a consensus definition of SPA

The absence of a universally accepted definition remains the most fundamental barrier to progress in the SPA field. External validation of SPA findings beyond the CARTaGENE cohort remains limited and should be prioritized in future research; replication in ethnically diverse populations and different healthcare settings will be essential to establish generalizability. Ethnic and racial differences in renin physiology and aldosterone regulation—such as the higher prevalence of low-renin states in individuals of African ancestry and differing salt sensitivity across East Asian populations—may influence SPA classification and should be considered in future validation studies (5, 18). Studies use varying renin cutoffs, different aldosterone assays, and divergent ARR thresholds, making cross-study comparison nearly impossible. Future research should determine whether consensus diagnostic criteria for SPA are feasible and clinically meaningful. However, renin-first phenotyping carries important limitations: renin measurement is subject to assay variability across laboratories, susceptible to interference from antihypertensive medications (ACEi, ARBs, beta-blockers, and diuretics), influenced by posture and salt intake, and population prevalence cutoffs have not been prospectively validated. The risk of overdiagnosis and labeling of individuals without established treatment pathways must be addressed before any screening program is considered. Standardized renin and aldosterone assays with harmonized reference intervals, and prospective validation of renin-first screening algorithms in diverse populations, are essential first steps (42). The 2024 ESC Class IIa recommendation for ARR-based screening in all hypertensive patients and recent Endocrine Society guideline simplification represent important steps, but dedicated SPA-specific criteria remain an unmet need (38, 41, 42).

### Prospective interventional trials

Recent longitudinal evidence further supports the concept that SPA and overt PA may represent evolving phenotypes rather than distinct entities (33). No prospective randomized trial has yet evaluated MRA intervention in SPA. Trials incorporating renin-first patient selection, hard endpoints (MACE, CKD progression), and validated surrogates (LVMI, albuminuria, PWV) are urgently needed. Novel biomarkers of MR signaling—including urinary extracellular vesicle proteins and mass spectrometry-based steroid profiling—may improve patient selection for such trials. Future consensus efforts should prioritize standardized assays and prospective cohort studies with pre-specified SPA definitions and hard clinical outcomes. This review is subject to the inherent limitations of a narrative methodology, and much of the available evidence derives from observational cohort studies in which residual confounding cannot be fully excluded.

## Conclusion

Subclinical primary aldosteronism has been proposed to represent a biologically plausible and potentially underrecognized segment of the aldosterone biology spectrum, with potential implications for cardiometabolic disease. SPA should currently be viewed as a research construct rather than a validated disease entity, and all clinical inferences should be interpreted within this context. The evidence reviewed here—from cellular mechanisms involving aldosterone-producing micronodules and somatic driver mutations, to population-based epidemiology demonstrating MACE risk below conventional PA diagnostic thresholds—collectively support reconceptualizing renin-independent aldosteronism as a spectrum rather than a binary diagnosis.

Observational evidence links SPA with increased arterial stiffness, adverse cardiac remodeling, major cardiovascular events, albuminuria, and incident hypertension, with effects not fully explained by blood pressure elevation alone and potentially reflecting MR-mediated pathophysiological effects. The conventional ARR-based screening paradigm, particularly when combined with minimum aldosterone thresholds, may under-recognize individuals within the subclinical spectrum. Evolving guidelines—including recent Endocrine Society criteria and the 2024 ESC recommendation for broader screening—provide partial solutions, but dedicated SPA diagnostic criteria remain an unmet need.

Therapeutically, the potential role of MRAs in SPA is hypothesis-generating: mechanistic rationale from overt PA treatment and large cardiorenal RCTs supports the concept, but prospective interventional evidence is entirely absent. SPA may challenge the current paradigm of PA case detection, and some individuals with low-renin profiles may be under-recognized by existing diagnostic frameworks. Future research should prioritize consensus diagnostic criteria evaluating renin-first phenotyping, prospective MRA intervention trials with hard cardiorenal endpoints, and integration of renin phenotyping into endocrine risk assessment.
