# Supplementary material for: Subclinical primary aldosteronism: toward a conceptual framework linking primary aldosteronism and low-renin hypertension
Source: Front Endocrinol (Lausanne). 2026 Jul 31;17:1899618. doi: 10.3389/fendo.2026.1899618 (PMC13473819; doi:10.3389/fendo.2026.1899618)
Supplement: Supplementary file 1 [file Table1.docx]

**Supplementary Table 1. Definitions of SPA or Low-Renin Aldosteronism Used Across Key Studies**

| **Study (Year)** | **Population** | **Renin criterion** | **Aldosterone criterion** | **ARR criterion** | **BP status** | **Key outcome** |
| --- | --- | --- | --- | --- | --- | --- |
| Brown et al. (2017) Ann Intern Med | 850 normotensive adults (MESA cohort) | PRA ≤0.5 ng/mL/h | Continuous variable | Continuous variable | Normotensive | HTN incidence 85.4/1,000 PY; aldosterone independently associated within suppressed-renin group (ref 9) |
| Hundemer et al. (2024) Circulation | 1,284 adults aged 40–69 years (CARTaGENE) | Renin ≤4 ng/L | Analyzed across ARR strata | ARR ≥70 pmol/L per ng/L | Hypertensive or normotensive | Arterial stiffness (PWV β=+0.33 m/s), LVMI (β=+5.1 g/m²), LVH (ref 7) |
| Goupil et al. (2025) Circulation | 1,284 adults aged 40–69 years (CARTaGENE) | Renin ≤4 ng/L | Analyzed across ARR strata | ARR ≥70 pmol/L per ng/L | Hypertensive or normotensive | HR 2.4 for MACE (95% CI 1.4–4.2); below PA diagnostic threshold (ref 8) |
| Shah et al. (2024) J Endocr Soc | 261 treatment-naïve hypertensives (primary care) | DRC <10 mU/L | Within normal range, variable | Variable | Hypertensive | 26.4% low-renin HTN; 18.0% confirmed PA; intermediate aldosterone phenotype (ref 14) |
| Markou et al. (2013) J Clin Endocrinol Metab | Predominantly normotensive cohort | Suppressed renin (PA biochemical profile) | Aldosterone within biochemical PA range (not formally measured separately) | ARR consistent with PA | Normotensive | OR 12.7 for HTN progression at 7-year follow-up (ref 17) |

*Abbreviations: ARR, aldosterone-to-renin ratio; BP, blood pressure; DRC, direct renin concentration; HTN, hypertension; LVMI, left ventricular mass index; LVH, left ventricular hypertrophy; MACE, major adverse cardiovascular events; PA, primary aldosteronism; PRA, plasma renin activity; PY, person-years; SPA, subclinical primary aldosteronism. Renin and aldosterone measurement methods vary across studies (PRA vs. DRC; immunoassay vs. LC-MS/MS), limiting direct comparability of criteria.*
